# Detection and Characterization of Circulating Tumor Associated Cells in Metastatic Breast Cancer

**DOI:** 10.3390/ijms17101665

**Published:** 2016-09-30

**Authors:** Zhaomei Mu, Naoual Benali-Furet, Georges Uzan, Anaëlle Znaty, Zhong Ye, Carmela Paolillo, Chun Wang, Laura Austin, Giovanna Rossi, Paolo Fortina, Hushan Yang, Massimo Cristofanilli

**Affiliations:** 1 Department of Medicine-Hematology and Oncology, Robert H Lurie Comprehensive Cancer Center, Feinberg School of Medicine, Northwestern University, Chicago, IL 60611, USA; giovirossi85@yahoo.it; 2 ScreenCell SA, Sarcelles 95200, France; benali@screencell.com (N.B.-F.); guzan@screencell.com (G.U.); aznaty@screencell.com (A.Z.); 3 Department of Medical Oncology, Sidney Kimmel Cancer Center, Thomas Jefferson University, Philadelphia, PA 19107, USA; Zhong.Ye@jefferson.edu (Z.Y.); Chun.Wang@jefferson.edu (C.W.); laustin@gmail.com (L.A.); hushan.yang@jefferson.edu (H.Y.); 4 Department of Cancer Biology, Sidney Kimmel Cancer Center, Thomas Jefferson University, Philadelphia, PA 19107, USA; carmela.Paolillo@jefferson.edu (C.P.); paolo.Fortina@jefferson.edu (P.F.); 5 Department of Molecular Medicine, University of Rome “Sapienza”, Rome 00185, Italy

**Keywords:** metastatic breast cancer (MBC), circulating tumor associated cells, circulating tumor cells (CTCs), circulating tumor cell clusters (CTC clusters), epithelial–mesenchymal transition (EMT), cancer associated macrophage-like cells (CAMLs), size-exclusion technology

## Abstract

The availability of blood-based diagnostic testing using a non-invasive technique holds promise for real-time monitoring of disease progression and treatment selection. Circulating tumor cells (CTCs) have been used as a prognostic biomarker for the metastatic breast cancer (MBC). The molecular characterization of CTCs is fundamental to the phenotypic identification of malignant cells and description of the relevant genetic alterations that may change according to disease progression and therapy resistance. However, the molecular characterization of CTCs remains a challenge because of the rarity and heterogeneity of CTCs and technological difficulties in the enrichment, isolation and molecular characterization of CTCs. In this pilot study, we evaluated circulating tumor associated cells in one blood draw by size exclusion technology and cytological analysis. Among 30 prospectively enrolled MBC patients, CTCs, circulating tumor cell clusters (CTC clusters), CTCs of epithelial–mesenchymal transition (EMT) and cancer associated macrophage-like cells (CAMLs) were detected and analyzed. For molecular characterization of CTCs, size-exclusion method for CTC enrichment was tested in combination with DEPArray™ technology, which allows the recovery of single CTCs or pools of CTCs as a pure CTC sample for mutation analysis. Genomic mutations of *TP53* and *ESR1* were analyzed by targeted sequencing on isolated 7 CTCs from a patient with MBC. The results of genomic analysis showed heterozygous *TP53* R248W mutation from one single CTC and pools of three CTCs, and homozygous *TP53* R248W mutation from one single CTC and pools of two CTCs. Wild-type *ESR1* was detected in the same isolated CTCs. The results of this study reveal that size-exclusion method can be used to enrich and identify circulating tumor associated cells, and enriched CTCs were characterized for genetic alterations in MBC patients, respectively.

## 1. Introduction

Breast cancer is the most common cancer in women and metastasis is the major cause of the mortality of patients [1]. The treatment for aggressive metastatic breast cancer (MBC) remains challenging as the intrinsic clinical subtypes, biological features of the metastases, and tumor heterogeneity including circulating tumor cells (CTCs) all contribute to the resistance to current standard strategies [2]. Current treatments mainly rely on expression profiles of the primary tumor or metastatic tumor biopsy. However, the expression profiles may change during or after adjuvant treatments or targeted therapies. A real-time tumor assay using a liquid biopsy that focuses on the analysis of CTCs and circulating tumor DNA (ctDNA) has been actively investigated as new therapeutic targets and drug resistance mechanisms in cancer patients [3,4]. Blood-based testing using a non-invasive and complementary approach has the potential to enhance therapeutic efficacy and improve the outcome of MBC patients.

Circulating tumor cells are rare cells that are shed into bloodstream from primary or metastatic tumors with the potential to initiate tumor metastasis in distant organs [5,6]. The enumeration of CTCs can provide consistent prognostic values in MBC patients [7,8]. The semi-automated CellSearch^®^ platform (Janssen Diagnostics, Raritan, NJ, USA) is the only FDA-approved platform for CTC detection and enumeration based on the expression of epithelial cell adhesion molecule (EpCAM) [7,8,9,10]. EpCAM-based assay is unable to detect CTCs with low or absent EpCAM expression and other circulating tumor associated cells, including stem cell-like tumor cells and CTCs undergoing epithelial–mesenchymal transition (EMT), which were identified by stem cell and EMT markers in early and metastatic breast cancer patients [11,12,13]; and cancer associated macrophage-like cells (CAMLs), which are specialized phagocytic myeloid cells found in the peripheral blood of patients with solid tumors including breast cancer, but not in healthy individuals [14]. CellSearch and other EpCAM-based assays may miss the detection of these cells.

Due to the limitation of EpCAM-based capture assays for the detection of various circulating tumor associated cells, many other technologies have been developed including size-execution enrichment devices [15]. ScreenCell^®^ technology is a range of specific devices and has been validated for the detection and molecular analysis of CTCs in several other solid tumors including prostate, pancreatic cancer, renal, colon, and lung [16,17,18,19,20,21]. Without surface-marker selection, size-execution method allows the capture of all types of circulating tumor associated cells in one blood draw and exploring the phenotypic and genetic heterogeneity of these cells.

Molecular characterization of CTCs is important to confirm their malignant origin and to identify the genomic abnormalities that may be undetectable or different from the primary tumor during disease progression and therapy resistance in order to achieve individual patient management and treatment. CTCs are rare and very heterogeneous populations of tumorigenic cancer cells in the blood of patients with metastatic cancer, which makes their isolation and characterization challenging. Isolation of pure CTCs from pre-enriched blood samples is a key step for exploring accurate genomic analysis. The DEPArray™ technology (Silicon Biosystems, San Diego, CA, USA) is the ideal system to collect pure CTCs for further molecular characterization. Recent studies showed a workflow that enables isolation and molecular characterization at single CTCs, which highlight the potential clinical utility for diagnostic molecular profiling in precision medicine [22,23,24]. The CTCs have been enriched from large population of red blood cells (RBCs) and white blood cells (WBCs) before single-cell sorting processing with DEPArray™ technology. CellSearch enrichment is the current commonly used method for DEPArray single-cell sorting.

In this pilot study, we aim to detect and validate circulating tumor associated cells including CTCs, CTC clusters, EMT CTCs, and CAMLs in one blood draw for MBC patients. In addition, we aim to explore the applicability of a protocol for the molecular characterization of pure CTCs by size exclusion enrichment method followed by single-cell sorting using the DEPArray™ in one patient blood sample. We target to analyze *TP53* and *ESR1* mutations based on the mutations data found in the corresponding patient′s tumor tissue sample.

## 2. Results

### 2.1. Detection of Circulating Tumor Associated Cells

**Figure 1:**
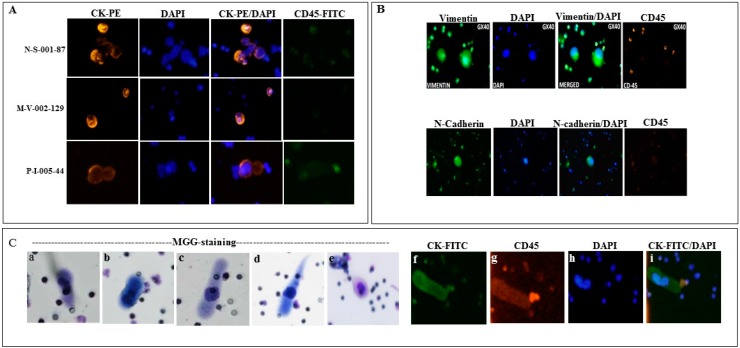
Detection of circulating cancer associated cells in metastatic breast cancer (MBC): (**A**) Representative immunostaining images from three patient samples with identifications (N-S-001-87, M-V-002-129, P-I-005-44) showing circulating tumor cells (CTCs) and CTC clusters with cytokeratin (CK) and 4′,6-diamidino-2-phenylindole (DAPI) positive and CD45 negative staining; (**B**) Immunostaining of epithelial–mesenchymal transition (EMT) CTCs showed mesenchymal markers Vimentin or *N*-Cadherin positive; (**C**) Representative images of cancer associated macrophage-like cells (CAMLs) from MGG (May-Grunwald Giemsa) and immunostaining, cytological phenotypes as: spindle-shaped (**a**,**d**); round (**b**,**e**); oblong (**c**); cytokeratin positive (**f**); CD45 positive (**g**); DAPI (**h**); and composite image of CK and DAPI (**i**). The images were acquired on microscope at 40× magnification.

Thirty patient blood samples were processed for the evaluation of CTCs and CTC clusters in this study. The morphological phenotypes of CTCs and CTC clusters are highly heterogeneous with cytokeratin (CK) positive staining variations in shape, size and degree of expression. Representative images from three patient samples are illustrated in Figure 1A. CTC-clusters were composed of two or more individual CTCs containing distinct nuclei bound together, and with intact cytoplasm membranes that distinguish the background staining from leukocyte marker CD45 staining. Among the 30 patients, CTCs were detected in 20 (66.7%) patients with a range from 1 to 347, and eight (26.7%) patients had at least one identified CTC clusters (≥2 CTCs) with a range from 1 to 10 (Table 1). CTCs represent very heterogeneous populations of tumorigenic cancer cells in MBC patient and some subpopulations had undergone EMT, which expressed decreased level or absence of epithelial markers including EpCAM. In order to characterize EMT phenotype on the captured CTCs, the immunostaining was performed with anti-Vimentin or *N*-Cadherin antibodies. EMT CTCs were identified as positive staining for Vimentin-Fluorescein isothiocyanate (FITC) or *N*-Cadherin-FITC, the representative images are shown in Figure 1B. In 28 processed patient samples, 13 (46.4%) patients had at least one detected positive either Vimentin or *N*-Cadherin positive CTCs (Table 1). Recent studies showed that CAMLs were found in blood of cancer patient and their presence was associated with the stage of disease as a blood based biomarker [14]. In this study, we detected CAMLs in MBC patient blood samples using May-Grunwald Giemsa (MGG) combined to immunofluorescence staining (Figure 1C). Like the CTCs, CAMLs are morphological heterogeneous in size ranges, shape, nuclear profiles. CAMLs express diffused cytoplasmic cytokeratin and CD45-positive signals. The results were consistent with previous observation [14], in which CAMLs express myeloid lineage (CD14^+^/CD11c^+^), CD45^+^, and both cytokeratin and EpCAM epithelial markers. Among the tested 20 patient samples, 15 (75%) patients had at least one detected CAMLs by either MGG or immunofluorescence staining (Table 1). Together, our results demonstrated that circulating tumor associated cells can be efficiently captured and enriched by the size-exclusion method for the cytological analysis, and a comprehensive test including multiple markers in one blood draw is necessary to explore the heterogeneity.

### 2.2. Enrichment, Single-Cell Sorting and Genome Amplification of Circulating Tumor Cells (CTCs)

**Figure 2:**
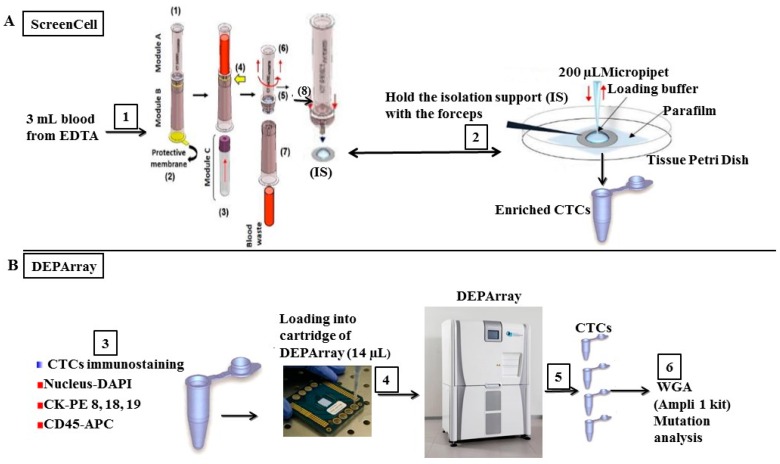
Overall processing workflow of blood sample on: ScreenCell^®^ Cyto device (**A**); and DEPArray™ platform (**B**). Step 1: 3 mL of blood was processed; Step 2: Cell suspension was recovered from the IS and transferred into 1.5 mL of Lo-bind eppendorf tube; Step 3: Enriched cells were stained by a manual staining procedure; Step 4: Cells were resuspended in a final volume of 14 µL and loaded in a DEPArray cartridge; Step 5: Sorting procedures were performed and single cells or pools of cells were isolated on DEPArray™ platform; Step 6: Isolated cells were subjected to WGA using the Ampli1 WGA kit and mutation analysis.

**Figure 3:**
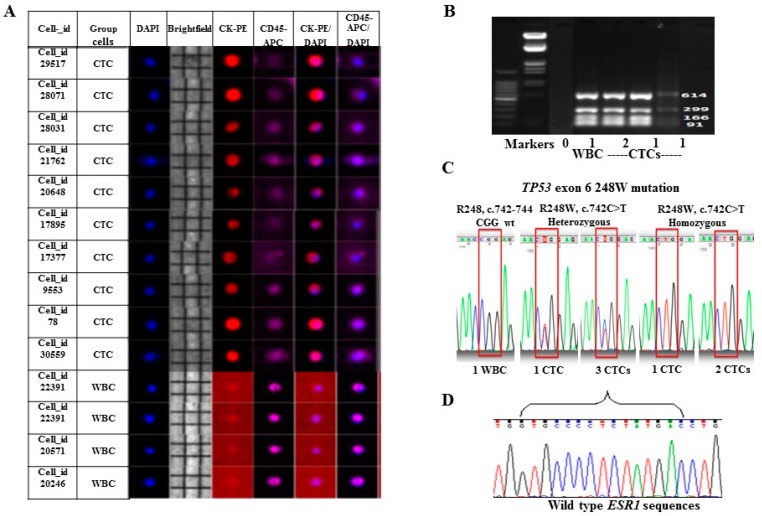
Genomic characterization of isolated CTCs. (**A**) Composite images visualized on the DEPArray™ platform after ScreenCell Cyto enrichment showing single CTCs (CK-PE^+^/DAPI^+^) and WBCs (CD45-APC^+^/DAPI^+^); (**B**) Gel images of Ampli1 quality control (QC) PCR products from CTCs and WBC showing the genome integrity with the presence of short, medium and long DNA fragments; (**C**) The *TP53* exon 6 R248W mutations were detected in single CTCs and pools of CTCs, wild-type sequences in single WBC; (**D**) Wild-type *ESR1* sequences were detected in all isolated CTCs.

To achieve the molecular characterization of the pure CTCs, a combined procedure is needed. The proposed workflow included several steps of CTC enrichment, single-cell sorting, and whole genome amplification (WGA) for sequencing is outlined in the Materials and Methods (Figure 2). One MBC patient sample SKM00447 was processed for the combined procedure. CTC enrichment from 3 mL of blood was first performed using ScreenCell^®^ Cyto devices followed by immunofluorescence staining using anti-CK, DAPI and anti-CD45 antibodies. Forty-six CTCs/3 mL of blood were identified with one additional device and immunostained CTCs. Ten single CTCs and four single WBCs with good images were identified and isolated on DEPArray™ system (Figure 3A). Single CTCs and pools of CTCs (two or three cells) were subjected to the WGA using Ampli1 WGA kit. Genome integrity was confirmed by PCR with the presence of short, medium, and long DNA fragments. Representative gel image from DNA amplification in some CTCs is shown in Figure 3B.

### 2.3. Mutation Analysis of TP53 and ESR1 in Isolated CTCs

The patient SKM00447 was metastatic inflammatory breast cancer with estrogen receptor (ER)-positive, progesterone receptor (PR)-negative, and HER2/neu-negative treated with multiple lines of systemic therapy, including endocrine therapy and combination chemotherapy. The mutation analysis of tumor tissue sample using commercially available NGS testing (FoundatioOne^®^, Foundation Medicine, Cambridge, MA, USA) showed that the tumor harbored *TP53* exon 6 R248W missense point mutation. Based on these clinical information, we analyzed single and pooled CTCs on the mutation status of *TP53* and *ESR1*, which is activating estrogen receptor1 and its mutation was associated with the resistance to endocrine therapy [25]. Interestingly, heterozygous *TP53* exon 6 R248W mutation was detected in one single CTC and pools of three CTCs, and homozygous *TP53* exon 6 R248W mutation was found in one single CTC and pools of two CTCs; one single WBC as control displayed the wild-type *TP53* sequence in exon 6 R248 (Figure 3C). The results suggest the heterogeneity of *TP53* mutations within the CTCs isolated even from same patient sample, and mutation profile of CTCs was consistent with the corresponding patient′s tumor tissue. In the same isolated CTCs, the most common mutations in exon 8 (c.1607-1621) of *ESR1* were not detected, the results showed same wild-type *ESR1* sequence from all CTCs (Figure 3D). *ESR1* mutation was also not found in this patient′s primary tumor test.

Overall, we demonstrated the applicable workflow using size-exclusion approach to enrich CTCs for pure CTC sorting on DEPArray™ platform and further molecular characterization analysis.

## 3. Discussion

CTC enumeration demonstrated a significant prognostic value, and additional blood based markers may also play roles in furthering prognostic and predictive values in MBC. Molecular characterization of CTCs can help determine the efficacy of selected therapeutic targets. In this pilot study, we isolated and analyzed multiple circulating tumor associated cells including CTCs, CTC clusters, EMT CTCs, and CAMLs by combining size-exclusion enrichment and cytological analysis in MBC patients. The CTCs can be efficiently enriched by size-exclusion method for further use in pure single-cells sorting on DEPArray™ platform. Mutational analysis of the isolated CTCs revealed a heterogeneous *TP53* mutation in one MBC patient.

The CellSearch™ system is the only CTC enumeration platform FDA-cleared and used in the clinical management of metastatic patients. Unfavorable prognostic outcome was determined based on a cut-off of ≥5 CTCs per 7.5 mL of blood in MBC [7,8,9,10]. However, there are no additional further prognostic markers in patients with ≥5 CTCs. Recent studies by our group and others have shown that the presence of CTC clusters when CTCs were enumerated at the same analyzing time on the CellSearch system might provide additional prognostic values over CTC enumeration alone for MBC patients [26,27,28]. The CTC clusters have shown much higher metastatic potential than single CTCs in breast cancer [27]. There is increasing evidence suggesting that EMT is involved in the metastatic process and drug resistance in breast cancer [29]. Clinical studies shown that EMT markers were detected in CTCs from early and metastatic breast cancer patients [13,30,31]. Despite these observations, very few studies have been able to demonstrate the independent prognostic value of EMT CTCs in MBC [32,33]. Currently, there are no standard reference markers and approved methods for the selection and detection of EMT CTCs. EpCAM-based assay is unable to capture EMT CTCs due to the loss of epithelial features in those cells, but CTCs expressing EMT and epithelial markers can be detected in breast cancer patients using the size-exclusion enrichment method [30,31]. Our results showed that all CTCs, CTC clusters and EMT CTCs can be detected and identified in the peripheral blood of MBC patients using the size-exclusion method. Additional studies in a larger number of patients are required to validate more EMT markers and the prognostic value in MBC.

Another potential blood-based biomarker for solid tumors are CAMLs. Recent studies have shown that the presence of CAMLs may be associated with malignant diseases and activation of innate immunity in cancer patients [14,34]. The detection of CAMLs may differentiate malignant disease and benign breast lesions as described in a recent report [35]. However, the function and prognostic value of CAMLs and the role in activation of innate immunity in cancer patients remain unknown. Consistent with previous findings, CAMLs were detected in 15 (75%) patients among 20 MBC patients and showed a considerable heterogeneity in the phenotypes. Given the larger size of CAMLs, size-exclusion methods may offer a significant advantage for capturing these cells. Further molecular and functional validation of CAMLs in larger numbers of patients will open a path to comprehensive blood-based biomarker access.

A recent clinical trial showed that CTC enumeration alone was not sufficient to guide chemotherapeutic treatment change for survival benefits in MBC [36], which highlights the importance of CTC molecular characterization to be more informative for the choice of treatment options. Monitoring CTC characterization over a period of time seems necessary since marker profiles can change during the course of therapy and disease progression. For the first time, we tested the feasibility that CTCs were enriched using the size-exclusion method and followed by DEPArray™ isolation to achieve mutation analysis on the single cell or pools of CTCs in one MBC patient sample. CTCs showed the matched *TP53* R248W mutation with the patient′s tissue tumor although a heterogeneity within CTCs. The results from one recent study showed the extreme heterogeneity of the mutational status of single CTCs in metastatic breast cancer patients [23], there is also a discordance between the mutational status of the primary tumor and CTCs among three analyzed patients. Enriched CTCs using the CellSearch method followed by DEPArray™ sorting for the molecular profiling of single CTCs has been recently reported [22,23,24]. Here, we present the size-exclusion method with high efficacy, low-cost, and flexibility to enrich CTCs. The method has a potential advantage for EMT CTCs and CAMLs enrichment to achieve the molecular profiling analysis in single cells or pure population of cells by combining DEPArray™ technology.

In summary, our data suggest that blood-based method using multiple markers analysis and molecular characterization of CTCs can be achieved by a combined method. Along with the further evaluation of association with patient outcomes, more studies with larger sample size and longer follow-up are warranted to confirm these findings and explore their clinical value. The comprehensive analysis from one blood draw as a non-invasive approach holds promise in clinical applicability for future personalize treatment of MBC patients.

## 4. Materials and Methods

### 4.1. Study Patients

Female MBC (stage IV) patients were identified from a clinic-based cohort of breast cancer patients who visited the Breast Care Center at the Sidney Kimmel Cancer Center at Thomas Jefferson University Hospital (Philadelphia, PA, USA). MBC diagnosis was based on tissue histology, complemented by radiological evaluations. This study was approved by the Office of Human Research, Division of Human Subjects Protection, Institutional Review Board (IRB) of Thomas Jefferson University Hospital (October 2013, protocol #13P.302). Written informed consent was obtained from all patients before blood samples were collected.

### 4.2. Blood Sample Collection and Processing

Approximately 8 mL of whole blood was collected in K2EDTA vacutainers or Transfix tubes and kept at room temperature. All samples were processed within 4 h (K2EDTA tubes) or 72 h (Transfix tubes) of being drawn. CTCs were captured on the isolation support (IS) using size-exclusion Cyto devices according to the manufacturer′s instruction (ScreenCell, Sarcelles, France) as previously described [16]. The ScreenCell^®^ device is holding an IS with 18-µm-thick polycarbonate membrane with circular pores (6.5 ± 0.33 µm) that are randomly distributed throughout membrane and a detachable nozzle attached to it. This nozzle guides the insertion of a collection tube to it to gently vacuum the blood through the IS, leaving the CTCs on the IS. The process is quick (3 min) [16]. Briefly, 3 mL of blood was mixed with 4 mL of ScreenCell^®^ FC2 dilution buffer and incubated for 8 min at room temperature. After incubation, diluted blood was passed through the ScreenCell Cyto device by vacuum force, and IS was rinsed by adding 1.6 mL of Phosphate Buffered Saline (PBS). After the enrichment, the IS was disassembled from the ScreenCell device, and allowed to air dry. The dried IS was stored at 4 or −20 °C until further cytological analysis. Two ISs were collected for each patient sample.

### 4.3. Cytological Analysis

For cytological evaluation, the IS was rehydrated with Tris-buffered saline (TBS) and subjected to standard MGG (May-Grunwald Giemsa) staining with May Grunwald dye and Giemsa (EMD Millipore, Billerica, MA, USA). Stained IS was evaluated based on the cytomorphological features under light microscope. MGG stained IS can be reused for further immunofluorescence staining after removing MGG staining by washing the IS with TBS containing 0.05% Tween 20 (TBST) for 20 min at room temperature (RT). Identification of CTCs and CTC clusters were determined based on the immunofluorescence staining of phycoerythrin (PE)-conjugated cytokeratins (CK-8, 18 and 19), DAPI (4′,6-diamidino-2-phenylindole) (CellSearch^®^ CTC kits, Janssen Diagnostics), and CD45-FITC antibodies (clone HI30, Biolegend, San Diego, CA, USA). In brief, the IS was rehydrated with TBS and permeabilized with TBS containing 0.2% Triton X-100. After blocking with 3% BSA (bovine serum albumin) for 30 min, the IS was stained with CK-PE and CD45-FITC for 45 min, and then DAPI for 5 min at RT. For EMT CTCs identification, the IS was stained with Vimentin-FITC (Santa Cruz Biotechnology, Santa Cruz, CA, USA) and *N*-Cadherin (eBioscience, San Diego, CA, USA) followed by Alexa Fluor 647 donkey anti-mouse antibody. The stained IS was viewed and images of stained cells were captured on Olympus BX43 Fluorescence Digital Imaging System or NIKON Eclipse 80i microscope. Positive CTCs were identified based on staining for both CK and DAPI positive, and for CD45 negative (CK^+^/DAPI^+^/CD45^−^). CTC clusters showed as a group of two or more individual CTCs containing distinct nuclei and intact cytoplasm membranes as previously described [26]. EMT positive CTCs were identified by positive staining for Vimentin or *N*-Cadherin and DAPI, and negative staining for CD45 (Vimentin^+^ or *N*-Cadherin^+^/DAPI^+^/CD45^−^). CAMLs were identified by MGG or immunofluorescence staining by CK-FITC.

### 4.4. CTCs Enrichment and Single-Cell Sorting by DEPArray™ System

CTCs were first enriched using ScreenCell Cyto devices and then sorted on DEPArray™ (Silicon Biosystems) system for collecting single CTCs or pools of CTCs. The overall experimental workflow is illustrated in Figure 2. CTCs enrichment was performed as described above and the single-cell sorting was conducted according to the manufacturer′s instruction for DEPArray™ analysis. Briefly, diluted blood (3 mL of blood mixed with 4 mL of FC2 dilution buffer) was passed through the hydrated IS of ScreenCell Cyto device with loading Buffer (PBS containing 3% BSA and 5 mM EDTA). The IS was covered with a drop of loading buffer after cell enrichment and was released from the device onto the pertri dish containing parafilm foil. The cells on the IS were detached by pipetting the liquid for several times and transferred into 1.5 mL of Lo-bind eppendorf tube. The recovering process was repeated by adding the loading buffer as necessary. The cells were centrifuged at 400× *g* for 10 min at RT and the supernatant was carefully removed. The cell pellets were reconstituted in TBS and proceeded to immunofluorescence staining by CK-PE, CD45-APC and DAPI (CellSearch^®^ CTC kits, Janssen Diagnostics). Enriched CTCs were then sorted on DEPArray™ platform (Figure 2). In brief, both 800 µL of SB115 buffer (Silicon Biosystems) and 14 µL of sample in SB115 buffer were loaded on the DEPArray™ cartridge chip (DEPArray™ A300K-cartridge, Silicon Biosystems). The chip was scanned in first step, and images of cells were captured and processed for selecting CTCs and WBCs based on CK-PE/DAPI positive staining for CTCs or CD45-APC/DAPI positive staining for WBCs as control. At final step, single CTCs or pools of CTCs, and single WBCs as control were recovered into individual 200 µL of PCR tubes. The samples were centrifuged at 14,000× *g* for 10 min and washed once with 100 µL of PBS. The buffer was removed from the tube and the cell pellets in 1–2 µL volume were proceeded to whole genome amplification (WGA) or stored at 80 °C until further WGA.

### 4.5. Ampli1™ Whole Genome Amplification and Quality Control Assays

The Ampli1™ WGA procedure is based on a ligation-mediated PCR following a site-specific DNA digestion with a library of highly concentrated DNA, which can be employed for further targeted genetic analysis. The DNA from the isolated CTCs or WBCs was amplified using the Ampli1™ kit WGA (Silicon Biosystems) according to the manufacturer′s instruction. Briefly, the isolated CTCs were thawed on ice and brought up to a volume of 1 µL of PBS to perform all the reaction in same tube as following steps: cell lysis, DNA digestion, ligation, and primary PCR according to the procedure of the supplier resulting in final volume 50 µL of WGA product. Genome integrity was evaluated using the Ampli 1-QC kit (Silicon Biosystems, Inc). Ampli 1-QC kit is a PCR assay which allows the simultaneous amplification of four different human genomic targets using 1 µL of an Ampli1™ WGA product. PCR products were visualized on a 1.5% agarose gel to determinate the genome integrity as previously described [22].

### 4.6. TP53 and ESR1 Mutations in CTCs

Amplified DNA from CTCs was used for *TP53* and *ESR1* mutations analysis which was performed by Sanger sequencing. The primers reverse 5′-GAAATCGGTAAGAGGTGGGC-3′ and forward 5′-GCTTGCCACAGGTCTCCCCA-3′ were used to amplify hot spot region in exon 6 of *TP53* (R248). The primers reverse 5′-CCCCAACCCATAGACTGAG-3′ and forward 5′-GCTAAGTGCTTTGGAGTTC-3′ were used to amplify the exon 8 of *ESR1* (K531E, V534E, P535H, L536R/Q, Y537N/C/S, D538G). PCR reactions were performed using the AmpliTaq-Gold Taq DNA polymerase (Applied Biosystems, Foster City, CA, USA) according to manufacturer′s instructions. Both genes were amplified at 95 °C for 30 s as the denaturation, at 60 °C for *TP53* and 57 °C for *ESR1* for 30 s as the annealing, and at 72 °C for 30 s as the primer extension for a total of 30 cycles. The amplified products were separated by electrophoresis on a 2% agarose/1× TAE (Tris, acetic acid, EDTA) gel. The PCR products were cleaned using the Amicon Microcon PCR purification kit (EMD Millipore, Billerica, MA, USA). The purified PCR product were finally sequenced on the ABI PRISM^®^ 3700 (Applied Biosystems) capillary genetic analyzer with the same primers in both directions.

## Figures and Tables

**Table 1:** Summary of CTCs, CTC-clusters, EMT CTCs, and CAMLs detected in MBC patients.

Circulating Tumor Associated Cells	Total Patients (N)	Positive Patients (%)
CTCs ≥ 1	30	20 (66.7)
CTC clusters ≥ 2 CTCs	30	8 (26.7)
EMT CTCs ≥ 1	28	13 (46.4)
CAMLs ≥ 1	20	15 (75.0)

MBC, metastatic breast cancer; CTCs, circulating tumor cells; CTC clusters, circulating tumor cell clusters; EMT, Epithelial–mesenchymal transition; CAMLs, cancer associated macrophage-like cells.
